# Status of research and development of pediatric vaccines for *Streptococcus pneumoniae*

**DOI:** 10.1016/j.vaccine.2016.03.107

**Published:** 2016-06-03

**Authors:** Mark R. Alderson

**Affiliations:** PATH, 2201 Westlake Avenue, Suite 200, Seattle, WA 98121, USA

**Keywords:** *Streptococcus pneumoniae*, Pneumococcal conjugate vaccine, Nasopharyngeal carriage, Protein vaccine, Infant vaccination

## Abstract

Pneumococcal disease is a major cause of morbidity and mortality in young children, particularly in the developing world. Vaccines are a critical strategy for protecting children from pneumococcal disease and licensed pneumococcal conjugate vaccines (PCVs) are having a significant impact on invasive pneumococcal disease and pneumococcal pneumonia throughout the world. Currently available PCVs do not, however, cover all pneumococcal serotypes and are complicated and relatively expensive to manufacture. While new PCV development is focused on either higher valency or more inherent affordability for developing countries, new vaccines are needed that offer serotype-independent protection. Vaccines containing proteins that are common to all pneumococcal serotypes could provide broad protection to children worldwide. Protein subunit and whole cell vaccines have advanced into Phase 1 and 2 clinical trials but face considerable challenges before they can become licensed and widely distributed.

## Highlights

• Pneumococcal conjugate vaccines (PCVs) have significantly reduced invasive pneumococcal disease (IPD) and pneumococcal pneumonia.
• PCV impact on IPD is blunted by serotype replacement.
• Pneumococcal protein vaccines may provide broad, serotype independent protection.

Pneumonia remains the leading global cause of death among children under age five, killing more than 900,000 children in 2013 and accounting for 15% of all child deaths [1]. *Streptococcus pneumoniae* (pneumococcus), the bacterium that is the most common cause of severe pneumonia, kills a half million children annually before their fifth birthday [2]. Pneumococcus also causes sepsis and meningitis and is one of the leading causes of bacterial otitis media (OM). In addition, pneumococcus causes significant morbidity and mortality in elderly adults [3]. Vaccines are a critical strategy for protecting children from pneumococcal diseases, particularly in Africa and Asia, where 95% of all pneumococcal deaths occur [5]. Pneumococcus has more than 90 serotypes, which vary by region. Currently licensed pneumococcal conjugate vaccines (PCVs) are effective, but protection is limited to pneumococcal disease caused by serotypes contained in the vaccines.

Invasive pneumococcal disease (IPD) is diagnosed by culture of pneumococcus from normally sterile sites, such as blood or cerebrospinal fluid. The majority of pneumococcal pneumonia, however, is difficult to diagnose because most cases are blood culture negative. A number of new diagnostic assays are in development, including molecular- and urine-based antigen tests. As pneumococci are transmitted by direct contact with respiratory secretions from patients and healthy carriers [4], it is important to differentiate disease from asymptomatic nasopharyngeal (NP) carriage in young children. Pneumococcal disease is treated with antimicrobials; however a number of pneumococcal strains have become resistant to first line antibiotics. Macrolide resistance has increased in many parts of the world and multidrug resistance has become a serious concern in the treatment of IPD, especially in Asian countries [6]. With the success of PCVs, however, fewer antibiotic-resistant pneumococcal infections are being reported since they tend to be the serotypes covered by the licensed vaccines.

## 1 Currently available vaccines and their limitations

Antibodies to the capsular polysaccharides on pneumococci are protective. Licensed PCVs are based on formulations of various capsular antigens derived from the selected serotypes. Successful vaccination in pediatric populations has been achieved by conjugating the polysaccharides to carrier proteins, which vary between manufacturers. In 2007, the WHO recommended the use of PCVs in all countries, setting highest priority for countries with high pneumonia and mortality rates in children less than five years of age [7]. Two licensed, WHO-prequalified PCVs are currently available: the 13-valent Prevnar 13^®^ manufactured by Wyeth Pharmaceuticals (Pfizer) and the 10-valent vaccine Synflorix^®^ manufactured by GlaxoSmithKline Biologicals (GSK). With the help of Gavi, the Vaccine Alliance, the Advance Market Commitment funding mechanism and other international donors, PCVs are being rolled out in low-income countries.

Prevnar 13^®^ and Synflorix^®^ are effective against vaccine serotypes but do not protect against all 90 plus pneumococcal serotypes. Despite a clear overall benefit of PCVs, increasing pneumococcal disease caused by non-vaccine serotypes, through serotype emergence or replacement, in high-income countries has been documented and may limit the overall benefit of PCVs [8]. This may be particularly relevant for low-income countries where there is a broader spectrum of serotypes that cause disease. Furthermore, PCVs are difficult to produce and relatively expensive as a result, which, without considerable financial assistance, limits their affordability and accessibility for low-income countries. Therefore, new vaccines are needed that are more affordable and provide either focused protection for children against serotypes prevalent in the developing world or, ideally, broad protection across all pneumococcal serotypes.

## 2 General approaches to vaccine development for low- and middle-income country markets

A number of approaches are being pursued to develop safe, affordable and effective vaccines against pneumococcal disease for children in the developing world. One such approach focuses on PCVs that protect against the most common serotypes causing IPD in low-income countries. Manufacturing processes are being designed to incorporate more efficient methods for fermenting and purifying polysaccharides, producing carrier proteins, conjugating polysaccharides to carrier proteins, and packaging vaccines into multi-dose vials, thereby reducing overall costs. Other strategies include targeting conserved surface epitopes common to most or all pneumococcal strains. A new generation of vaccines is targeting common proteins including, but not limited to, pneumolysin, pneumococcal surface protein A (PspA), pneumococcal surface protein C (PspC), pneumococcal surface antigen A (PsaA), neuraminidase enzymes, and histidine-triad proteins, with the aim of inducing broader cross-serotype protection than current PCVs. In addition, potentially low-cost, vector-based technologies are allowing for the expression of pneumococcal proteins in attenuated *Salmonella* strains. Preclinical studies have demonstrated that vaccines based on common pneumococcal proteins can protect mice from NP carriage, pneumonia, and IPD after challenge with *S. pneumoniae*. The most advanced protein vaccine candidates have been tested clinically, including Phase 2 trials in infants and young children. Clinical data will be forthcoming over the next few years regarding the ability of protein vaccines to protect against NP carriage, OM, lower respiratory tract infections, and pneumonia. Additional strategies that also hold promise for enabling low-cost and broadly protective vaccines include inactivated whole cell preparations and vaccines that combine protein and conjugate technologies. The latter approach involves either the addition of proteins to an existing PCV or the use of common pneumococcal proteins as the carrier for a PCV. To be deemed successful, protein subunit vaccines must demonstrate equivalence with licensed vaccines in their ability to reduce both pneumococcal disease and NP carriage rates, which will in turn confer greater herd immunity. Protein vaccines that impact disease but not NP carriage would likely be used in conjunction with PCVs (Table 1).

## 3 Technical and regulatory assessment

The first PCV—the 7-valent Prevnar^®^—was licensed in 2000 based upon data from a large US clinical trial that showed efficacy against IPD. Incidence of IPD overall and for PCV-7-serotypes declined by 45% and 94%, respectively [9]. Rates of pneumonia and OM also decreased. The WHO developed recommendations for the production and control of PCVs to provide licensure criteria for new PCVs [10]. Second-generation PCVs—including Synflorix^®^ and Prevnar 13^®^—were licensed on the basis of non-inferiority to PCV-7 in immunogenicity studies rather than efficacy trials. Immunogenicity was assessed by serotype-specific immunoglobulin G antibody concentrations as measured by enzyme-linked immunosorbent assay and functional antibody levels as measured by opsonophagocytic assay. Other PCVs in development will likely follow the same licensure pathway used by Synflorix^®^ and Prevnar 13^®^, except that they may be required to use one of these vaccines as a comparator for non-inferiority trials. The protein subunit vaccines under development face several challenges to licensure. Correlates of protection capable of predicting clinical benefits may be necessary for the licensure of protein vaccines, but are not yet well-defined. Whether or not a vaccine can be licensed on the basis of its impact on NP carriage alone and/or OM is unclear. Or would a full pneumonia or IPD efficacy trial be needed? Market approval for an OM indication may make performing post-marketing studies for pneumonia and/or IPD possible. Advancing to WHO prequalification rapidly once licensure is obtained is critical since the disease burden is highest in countries with the greatest resource constraints.

## 4 Status of vaccine R&D activities

As noted above, a number of vaccine manufacturers in low and middle income countries are engaged in the development of multivalent PCVs. Most of these efforts are at the preclinical stage of development and vaccines are being manufactured to approximate currently licensed PCVs in most respects but at a lower cost [11]. The most advanced candidates that are in clinical development (Phase 1 and 2 trials) are 10- to 13-valent PCVs being developed by the Serum Institute of India, Ltd., SK Chemical Co., and Panacea Biotech, Ltd.

Other pneumococcal vaccine development efforts are focused on conserved epitopes on proteins common to all pneumococcus serotypes. Sanofi Pasteur is developing the most advanced protein-subunit vaccine, which comprises the following recombinant proteins: pneumococcal histidine triad protein D (PhtD), pneumococcal choline-binding protein A (PcpA), and pneumolysoid [12]. This trivalent vaccine has completed a Phase 1 age de-escalation study in Bangladesh, demonstrating both safety and immunogenicity. An alternative approach that PATH is pursuing in collaboration with Boston Children's Hospital is an inactivated pneumococcal whole cell vaccine candidate that may provide broad protection and be inexpensive to produce and administer. This vaccine candidate is intended to protect against both NP carriage and invasive disease/pneumonia and is currently in a Phase 1/2 age de-escalation clinical trial in Kenya after completing a Phase 1 study in healthy adults in the United States. Genocea Biosciences is developing a trivalent protein vaccine intended to protect against NP carriage. A Phase 1 clinical trial of this product has completed and found the vaccine candidate to be safe and immunogenic. It is currently being assessed for an impact on carriage in an adult experimental NP challenge model.

GSK is developing a bivalent protein vaccine, comprising PhtD and pneumolysoid that is designed to add to the protection provided by PCVs. In collaboration with PATH, the Medical Research Council and the London School of Hygiene and Tropical Medicine, GSK is testing its protein-plus-conjugate vaccine candidate in a Phase 2 clinical trial in The Gambia. Results could shed light on the potential for protein-based approaches to generate more protective pneumococcal vaccines for young children. In another approach, Liquidia Technologies is using its particle-based technology that combines polysaccharides and common proteins in order to mimic conjugate vaccines. Affinivax is also using its multiple antigen presenting system technology to couple polysaccharides with proteins [13].

## 5 Major advances in last 3–5 years

The licensure of the higher-valency PCVs (10- and 13-valent) and their recent introduction into Gavi-eligible countries is beginning to have a significant global impact on pneumococcal disease. Vaccine manufacturers in low- and middle-income countries are working to develop and license additional PCVs that would be available at a lower cost than current PCVs. Clinical development of protein subunit vaccines has advanced considerably, such that multiple candidates have entered into Phase 2 trials that include NP carriage in infants as an outcome. Several vaccine developers including GSK, Sanofi Pasteur, and Genocea are actively advancing protein subunit vaccines.

## 6 Likelihood for financing

Gavi has supported the rollout of Prevnar 13^®^ and Synflorix^®^ in more than 25 countries since 2010. More than 50 countries have been approved for Gavi support to introduce PCVs into their national immunization programs. The WHO recommends a three-dose schedule for PCVs (either a three-dose schedule in infants or a two-dose prime-plus-booster dose). The current cost to Gavi is approximately US $3.10–$3.40 per dose or $9.30–$10.20 per fully vaccinated child. To date, Gavi and its partners have supported the immunization of more than 10 million children. PCV procurement represents a large financial burden for Gavi and is responsible for more than 40% of Gavi's expenditures on vaccines. Lower cost pneumococcal vaccines, particularly those that could also increase coverage against pneumococcal serotypes, are of highest importance.  

*Conflict of interest statement*: None declared.

## Figures and Tables

**Table 1:** Development status of current vaccine candidates (POC = proof of concept trial).

Candidate name/identifier (company)	Preclinical	Phase 1	Phase 2	POC	Phase 3
*Pneumococcal conjugate vaccines (PCVs)*					
15 valent PCV (Merck & Co.)			X		
12 valent PCV (GlaxoSmithKline [GSK])			X		
10 valent PCV (Panacea Biotech, Ltd.)			X		
10 valent PCV (Serum Institute of India, Ltd.)			X		
Multivalent PCV (SK Chemicals and Sanofi Pasteur)	X				
Multivalent PCV (Pnuvax)	X				

*Common protein vaccines*					
Pneumococcal whole cell vaccine (PATH/Boston Children's Hospital)			X		
PhtD/pneumolysoid/PcpA common protein vaccine (Sanofi Pasteur)			X		
Trivalent protein (Genocea Biosciences)		X			
Two or more pneumococcal proteins loaded on bacterium-like-particles (Mucosis B.V.)	X				
Live Recombinant Attenuated Salmonella Vaccine (Arizona State University)		X			

*PCVs incorporating common proteins*					
PhtD/pneumolysoid common protein vaccine (GSK)			X		
Particle based PCV (Liquidia Technologies)	X				
Novel conjugation technology using common protein carriers (Affinivax, Inc.)	X				
